# 

**Published:** 1904-10

**Authors:** 


ESTABLISHED IN 1839

THE OLDEST DENTAL MAGAZINE IN THE WORLD

INDEPENDENT---------PUBLISHED BY DENTISTS FOR DENTISTS

VOLUME XXXV
NUMBER 10
THIRD SERIES
OCT.
1904

EDITOR:

WM. GIRD BEECROFT, D. D. S., Madison, Wis.

ASSOCIATE EDITORS:

ALBERT H. KETCHAM, D. D. S.. Denver, Colo

EMORY A. BRYANT, D. D S., Washington, D. C.

GEO. S MARTIN, D. D. S., L. D. S Toronto Junction, Ontario.

Publisheu on the 10th day of each month.

Subscription Price, $1.00 per year.

Foreign Countries, $1.50 pe year.

Address all communications, both business and editorial to the editor

**Entered as Second Class Matter, Madison, Wis.**

